# Performance of a DNA methylation marker panel using liquid-based cervical scrapes to detect cervical cancer and its precancerous stages

**DOI:** 10.1186/s12885-018-5125-8

**Published:** 2018-12-03

**Authors:** Martina Schmitz, Kristin Eichelkraut, Dana Schmidt, Ilona Zeiser, Ziad Hilal, Zena Tettenborn, Alfred Hansel, Hans Ikenberg

**Affiliations:** 1 oncgnostics GmbH, Winzerlaer Strasse 2, Jena, Germany; 2 MVZ CytoMol, Berner Str. 76, Frankfurt, Germany; 3 ZyDoLab, Institute for Cytology and Immune Cytochemistry, Markt 10, Dortmund, Germany

**Keywords:** DNA methylation, Human papillomavirus (HPV), Biomarkers, Epigenetic markers, Cervical cancer

## Abstract

**Background:**

A change of cervical cancer screening algorithms to an HPV-based screening setting is discussed in many countries, due to higher sensitivity of HPV testing compared to cytology. Reliable triage methods are, however, an essential prerequisite in such a setting to avoid overtreatment and higher screening costs.

**Results:**

In this study, a series of cervical scrapes collected in PreservCyt liquid-based cytology (LBC) medium from women with cervical cancer (*n* = 5), cervical intraepithelial neoplasia grade 1–3 (*n* = 74), and normal cytology (*n* = 201; further *n* = 352 collected in SureThin®) were assessed for methylation of the marker regions ASTN1, DLX1, ITGA4, RXFP3, SOX17, and ZNF671 using the GynTect assay and compared to cobas® HPV and CINtec Plus® biomarker results. All samples from women with cervical cancer, 61.2% of CIN3, 44.4% of CIN2 and 20.0% of CIN1 cases were scored positive for the GynTect methylation assay. In contrast, all CIN, irrespective of severity grade, and carcinomas were positive by both, CINtec Plus and cobas HPV. The specificity of GynTect for CIN3+ was 94.6% compared to 69.9% for CINtec Plus and 82.6% for cobas HPV (all HPV types) and 90.6% for cobas HPV 16/18. DNA methylation analysis of this methylation marker panel (GynTect assay) in cervical scrapes consistently detects cervical cancer and the majority of CIN3 as well as a subset of CIN1/2 lesions. The detection rate among cytologically normal samples is extraordinarily low (1.5%).

**Conclusion:**

GynTect shows excellent performance when using cervical scrape material collected in liquid-based cytology media, a prerequisite for employing such a test as a triage in screening programs. Compared to the other test systems used in this work, GynTect showed higher specificity while still detecting all cancer cases.

## Background

With the availability of screening programs, cervical cancer incidence and mortality have markedly decreased, especially in developed countries [1]. The effects of the cytology-based diagnostics – the so-called Pap test, the most prominent screening tool applied even nowadays – have, however, levelled-off the last decade, mainly because of the limited sensitivity for precancerous lesions, as well as limited participation of the women. On the other hand, limited specificity of the Pap smear also leads to over-diagnosis and over-treatment, mainly among young women. Therefore, alternative screening tools and algorithms, which may help to overcome these limitations of cytology have been evaluated in the recent years.

Testing for high-risk human papillomaviruses (hrHPV), the sexually transmitted infectious agents that evoke cervical cancer, could substantially improve the sensitivity of screening [2]. Thus, in several countries HPV testing has already been implemented (e.g. the Netherlands, USA) or is being implemented in screening (e.g. Germany, New Zealand) [3]. Infection with one of the high-risk HPV types is a prerequisite for the development of nearly all cervical cancers. HPV screening has high sensitivity, but lacks, however, specificity, since most women infected with HPV will clear such an infection without developing lesions [4]. Therefore, HPV-based cervical cancer screening only makes sense with the availability of triage methods that allow the detection of precancerous lesions and cancer cases among women tested HPV-positive [5].

CINtec Plus® (Roche Diagnostics, Mannheim, Germany), which detects the two biomarkers p16 and Ki-67 simultaneously by immunocytochemistry, has been assessed as a triage test for HPV-positive women in several studies [6, 7] . In a primary screening setting, CINtec Plus® shows an increase in sensitivity for CIN2+, at noninferior specificity, over Pap testing [7]. In a triage setting using HPV testing as primary screening, however, a very specific second-line test is necessary in order to further reduce colposcopy referral.

In this context, hypermethylation of certain DNA regions during the course of carcinogenesis may provide a promising tool for triage of a highly sensitive screening, which finds virtually all disease cases, but lacks specificity, as is the case if testing for HPV [8, 9]. DNA methylation patterns change in numerous diseases, among these also cancer [10]. DNA methylation patterns strongly associated with cancer phenotypes may therefore be used for different aspects of cancer diagnostics [11]. In cervical cancer a DNA hypermethylation marker panel consisting of the six marker regions ASTN1, DLX1, ITGA4, RXFP3, SOX17, and ZNF671 may be a useful tool for triaging HPV-positive women [12, 13]. A diagnostic test comprising these six markers has been developed under the name GynTect. It received CE IVD mark in September 2015 [13], confirming the assay quality in a diagnostic setting using cervical smears collected in Specimen Transport Medium (STM, Digene HPV test, QIAGEN). In-between GynTect has been adapted to the use on cervical smears collected in liquid-based cytology (LBC) media. These have the advantage that cervical smears can be stored, so triage after an abnormal cytology and/or positive HPV test can be performed from the same sample, a feature that would be the prerequisite for HPV-based cervical cancer screening with triage from the same sample.

In the present work we investigated the performance of GynTect in comparison to cobas HPV 16/18 genotyping and CINtec Plus. Especially the latter assay is also discussed for triaging abnormal screening results.

## Methods

### Patient samples

Residual samples from routine cervical cancer screening (cytology in first line with HPV testing in case of abnormal cytology results) with normal cytology findings (NILM) as well as residual samples from patients with abnormal histopathology findings (CIN1, CIN2, CIN3, cervical cancer), all available in liquid-based cytology (LBC) media, were used for the study. This cohort based on PreservCyt® (Hologic, Wiesbaden, Germany) samples (collected at a commercial laboratory (CytoMol, Frankfurt, Germany)) consisted of 201 anonymized screening samples from women with normal cytology (NILM, determined by liquid based cytology using the computerised ThinPrep Imager), and 79 anonymized screening and triage samples from women with histopathology diagnosis CIN1 (*n* = 5), CIN2 (*n* = 19), CIN3 (*n* = 50), and cervical cancer (*n* = 5).

To further determine the specificity of the GynTect assay, 352 LBC samples collected in SureThin® media (CytoGlobe®, Burgdorf, Germany) from routine screening were tested using GynTect. All samples had normal cytology (NILM) and were collected in the commercial laboratory ZyDoLab, Institute for Cytology and Immune Cytochemistry, in Dortmund, Germany.

### DNA methylation marker analysis

For performing the GynTect assay, samples were processed as described in the instructions for use. Briefly, LBC samples were vortexed for a few seconds, and 1 ml of each sample to be used for the assay was immediately transferred into a 2 ml Eppendorf tube. Cells were centrifuged, the supernatant removed and the pelleted cells were resuspended using 40 μl of GynTect lysis buffer and incubated at 60 °C for 30 min at 1000 rpm in a thermoshaker (Thermomixer, Eppendorf, Germany). 40 μl of the incubated material was directly used for bisulfite treatment using the EpiTect® Fast Bisulfite Kit (Qiagen, Hilden, Germany). After elution of the bisulfite-converted DNA with 20 μl water, 70 μl water was added and 10 μl was applied to each single reaction in the GynTect real-time methylation-specific PCR (qMSP) assay as described elsewhere [13]. The qMSPs were run on a 7500 Real-Time PCR system (Life technologies; Thermo Scientific, USA) analysing the 6 methylation markers ASTN1, DLX1, ITGA4, RXFP3, SOX17, and ZNF671, and two controls for each sample, a DNA quality control (ACHE) and a methylation control (IDS), each in a separate tube. In addition, a positive control for determining the PCR quality was included in each PCR run. It had to show positive signals for each methylation marker and control marker. A “no template” control using water instead of template was also included in each qMSP run.

For each marker the Ct-value was determined and a delta Ct was calculated between the Ct-value of the quality control marker ACHE and the Ct value for each marker. To be scored positive, the delta Ct has to be ≤9 for ASTN1, DLX1, ITGA4, RXFP3, SOX17 and ≤ 10 for ZNF671. Each marker has a score if positive (DLX1 0.1; ASTN1, ITGA4, RXFP3, SOX17 each 0.2; and ZNF671 0.5) and GynTect is considered to be positive if the total GynTect score is equal or higher than 0.5. To be scored valid, the Ct value for the control marker ACHE had to be ≤32 for the respective sample.

### Cytology, HPV testing and p16/Ki67 immunocytochemistry

To determine the cytology status, PreservCyt® samples were analysed using computer-assisted cytology at CytoMol. The 352 SureThin samples were analysed by two experts experienced in morphological diagnostic at the cytological laboratory ZyDoLab in Dortmund, Germany.

The cobas® HPV test (cobas z 4800 system) and CINtec Plus® were in most cases performed during routine testing at the molecular diagnostics laboratory CytoMol in Frankfurt, Germany. For samples with missing HPV and/or CINtec Plus results, testing was done subsequent to GynTect testing. HPV testing was done for all samples, CINtec Plus® was performed for all CIN and cancer samples and a proportion of the NILM samples (*n* = 59).

## Results

In the study, GynTect showed an excellent performance. Of the 280 ThinPrep LBC samples included, only four (=1.4%) yielded a “not valid” result due to a too high Ct value for the ACHE control marker.

All five carcinomas included in the study were scored GynTect-positive. Of the 49 valid samples with histopathology-confirmed CIN3, 30 (= 61.2%) turned out to be GynTect-positive, whereas eight of the 18 valid CIN2 samples (= 44.4%) and one of the five CIN1 samples were GynTect-positive (see Table. 1). Regarding the NILM group of this cohort, three (1.5%) of the 199 valid samples showed a positive GynTect result (Table 1).

**Table 1:** Detection rates of the GynTect assay for the sample cohort in PreservCyt (Hologic)

All ages	CxCa (*n* = 5)	CIN3 (*n* = 50)	CIN2 (*n* = 19)	CIN1 (*n* = 5)	NILM (*n* = 201)
# tested samples	5	50	19	5	201
# GynTect positive	5	30	8	1	3
# GynTect negative	0	19	10	4	196
# GynTect invalid	0	1	1	0	2
# valid samples	5	49	18	5	199
detection rate [%]	100.0%	61.2%	44.4%	20.0%	1.5%

Regarding different age groups (see Table 2), detection rates increased with age (not significant), except for cancer due to a 100% detection rate irrespective of the age. For CIN 1 and 2 due to the very small number of samples this is only a tendency.

**Table 2:** Detection rates for the GynTect assay in different age groups. Especially the NILM group is very imbalanced between the age groups

**<**35y	CxCa (*n* = 2)	CIN3 (*n* = 22)	CIN2 (*n* = 5)	CIN1 (*n* = 3)	NILM (*n* = 38)
# tested samples	2	22	5	3	38
# GynTect positive	2	12	2	0	0
# GynTect negative	0	9	3	3	37
# GynTect invalid	0	1	0	0	1
# valid samples	2	21	5	3	37
detection rate [%]	100.0%	57.1%	40.0%	0.0%	0.0%
≥ 35y	CxCa (*n* = 3)	CIN3 (*n* = 28)	CIN2 (*n* = 14)	CIN1 (*n* = 2)	NILM (*n* = 163)
# tested samples	3	28	14	2	163
# GynTect positive	3	18	6	1	3
# GynTect negative	0	10	7	1	159
# GynTect invalid	0	0	1	0	1
# valid samples	3	28	13	2	162
detection rate [%]	100.0%	64.3%	46.2%	50.0%	1.9%

**Fig. 1:**
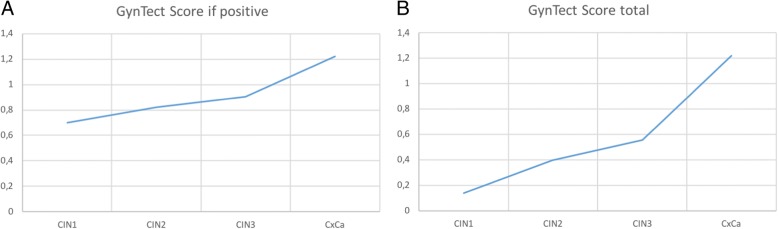
GynTect score for **a**) all positively tested samples and **b**) for the total number of samples tested with GynTect

The number of markers positive in each sample is increasing with the severity of the histopathological finding. Of the five cancer cases one sample was positive for 4 GynTect markers, three for 5 GynTect markers and one for all 6 GynTect markers, resulting in a mean value of 5 markers being positive for the cancer cases. In the CIN3 and CIN2 group, the mean values were 3.5 and 3 markers, respectively. The CIN1 sample tested positive was positive for two GynTect markers. This is also reflected in the GynTect score within the histopathology groups, which is shown in Fig. 1.

The detection rate of CINtec Plus was 100% for all valid CIN and cancer cases (two invalid samples: one cancer sample and one CIN2 sample). In the NILM group tested with CINtec Plus (*n* = 59), one sample was tested positive for CINtec Plus and 3 samples were invalid. The cobas HPV test was positive for all CIN and cancer samples except two CIN3 samples, which were scored HPV-negative. In the NILM group, 15 (8.0%) of the 201 samples were positive for the cobas HPV test, one sample was invalid. Using HPV16/18 genotyping, the false-positive rate in the NILM group decreases to 3.0%. One cancer case, however, which was not HPV16- or HPV18-positive, would have been missed. All CIN lesions showed 16/18 positivity rates between 60.0% (CIN1) and 66.0% (CIN3).

The three samples within the NILM group tested GynTect-positive, were HPV- and CINtec Plus-negative. The CINtec Plus-positive sample within the NILM group was also tested positive with cobas HPV (HPV18-positive), all other HPV-positive samples were GynTect- and CINtec Plus-negative.

**Fig. 2:**
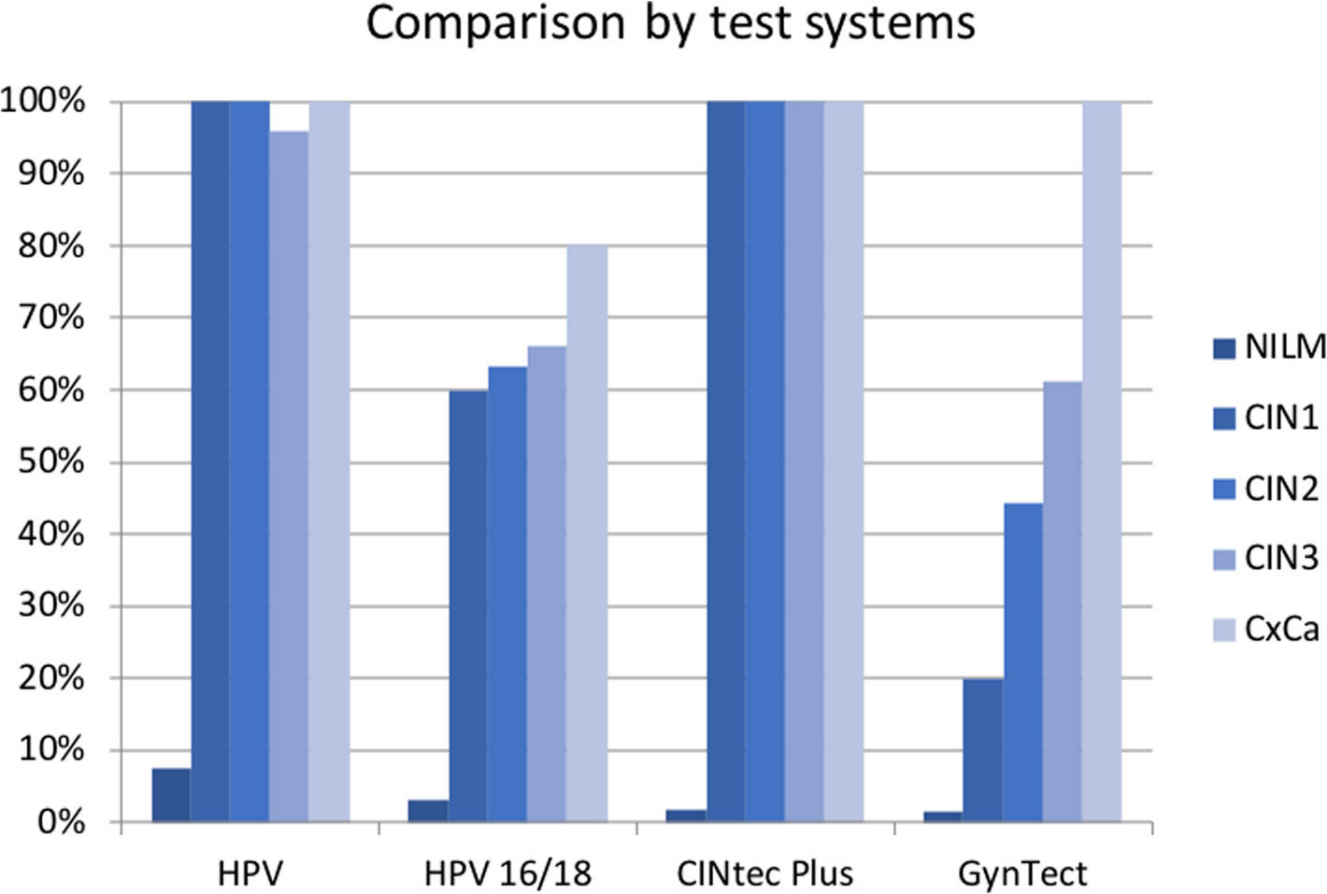
Performance of the different test systems

Comparing the three different test systems, GynTect shows an increase in the detection rate with severity of the CIN lesion. Not only the percentage of samples scored positive within, but also the average GynTect score itself between the histopathology groups, increased, respectively. In contrast, cobas HPV and CINtec Plus do not differentiate between CIN 1, 2, 3 lesions or cancer cases (Fig. 2 and Table 3). Two CIN 3 samples tested negative for cobas HPV are the only exception. Both HPV-negative CIN 3 samples were also GynTect-negative.

**Table 3:** Detection rates of the different test systems and rates of invalid test results

	cobas HPV (all 14 types)	cobas HPV (HPV16/18)	CINtec Plus	GynTect
NILM	7.5%	3.0%	1.8%	1,5%
CIN1	100.0%	60.0%	100.0%	20.0%
CIN2	100.0%	63.2%	100.0%	44.4%
CIN3	96.0%	66.0%	100.0%	61.2%
Cancer	100.0%	80.0%	100.0%	100.0%
invalid test results	0.36%	0.36%	3.62%	1.43%

For each test system, sensitivity, specificity, PPV and NPV were calculated referring to histopathology (cytology for the NILM group) findings (Table 4). GynTect demonstrated a sensitivity for CIN3+ of 64.8% at a specificity of 94.6%. For CIN2+, a sensitivity of 59.7%, with 98% specificity, was obtained. Cobas HPV 16/18 genotyping achieved a sensitivity for CIN3+ and CIN2+ of 67.3 and 66.2% and a specificity of 90.6 and 95.5%, respectively. Of note is that one cancer case would have been missed using only HPV16 and 18. Cobas HPV for all types is more sensitive but shows a rather low specificity.

**Table 4:** Sensitivity, specificity, PPV and NPV of the different test systems regarding the detection of CIN2+ and CIN3+

	cobas HPV (all 14 types)	cobas HPV (HPV16/18)	CINtec Plus	GynTect
Sensitivity				
CIN2+	97.3%	66.2%	100.0%	59.7%
CIN3+	96.4%	67.3%	100.0%	64.8%
Cancer	100.0%	80.0%	100.0%	100.0%
Specificity				
CIN2+	90.2%	95.6%	90.2%	98.0%
CIN3+	82.6%	90.6%	69.6%	94.6%
PPV				
CIN2+	78.3%	84.5%	92.3%	91.5%
CIN3+	57.6%	63.8%	69.2%	74.5%
NPV				
CIN2+	98.9%	88.7%	100.0%	87.3%
CIN3+	98.9%	91.9%	100.0%	91.7%
invalid tests	0.36%	0.36%	3.62%	1.43%

CINtec Plus showed a 100% sensitivity for both, CIN2+ and CIN3+ samples in this cohort, its specificity is not directly comparable to GynTect and cobas HPV due to less NILM samples tested with CINtec Plus.

Looking at the predictive values, GynTect demonstrated a PPV of 74.5 and 91.5% for CIN3+ and CIN2+ and an NPV of 91.7 and 87.3%. Cobas HPV 16/18 showed a PPV of 63.8 and 84.5% and an NPV of 91.9 and 88.7% for CIN3+ and CIN2+, respectively. Cobas HPV for all types shows higher NPVs (98.9% for both, CIN2+ and CIN3+) along with lower PPVs (78.3 and 57.6% for CIN2+ and CIN3+, respectively) compared to genotyping. NPV and PPV for CINtec Plus are not directly comparable to GynTect and cobas HPV due to less NILM samples, as already mentioned above.

To confirm the specificity of GynTect for NILM samples, 352 samples from routine screening collected in SureThin liquid-based cytology medium (CytoGlobe, Burgdorf, Germany) were tested. Of the 352 samples, only 6 were invalid (1.7%), and of the valid 346 samples 3 were tested positive with the GynTect assay, achieving a specificity of 99.1% in this sample group.

## Discussion

In previous studies we have shown that hypermethylation of CpG islands in proximity to the genes DLX1, ITGA4, RXFP3, SOX17, and ZNF671 correlated with the presence of cervical precancerous lesions and cervical cancer [12]. The molecular diagnostic test GynTect based on these results was designed and developed to allow detection of these marker regions by standard methods in cervical smears collected in the denaturing specimen transport medium (STM), which is originally used for QIAGEN’s Digene HPV test [13]. Utilization of this medium has, however, its limitations, the most important being that from STM only molecular tests but no cytology can be performed. Furthermore, DNA stability is limited in STM. In contrast, cervical smear material collected in liquid-based cytology media can be used more flexibly. As a main advantage, the cellular material preserved in this medium can be used for cytology as well as for molecular biology tests. This enables the performance of triage tests from the sample taken for the initial screening test, a feature which is increasingly demanded as a prerequisite for diagnostics [14].

In this study we evaluated whether GynTect is suitable for using residual material from liquid-based cytology samples and thus fulfils this prerequisite. Furthermore, we compared the results with cobas HPV testing including genotyping for HPV 16/18, and CINtec Plus data obtained from the same samples. The latter test is also discussed as triage test for patients with abnormal cytology or positive HPV tests.

GynTect might provide the possibility to test if a woman with an abnormal cytology finding in the Pap smear and/or a positive HPV test result, has a precancerous lesion that requires follow-up and treatment. For this purpose we used samples for which the cytology findings and, for all Pap-abnormal cases, the histopathology results were available.

GynTect showed a very good technical performance, since among all 280 PreservCyt samples only 4 (1.4%) and among the 352 SureThin samples, only 6 (1.7%) were tested invalid with GynTect.

The excellent performance of GynTect from LBC samples was directly visible in the Ct values obtained for the control DNA regions detected in the test system. In many samples less than 28 cycles were observed as Ct value for the control markers included in the test, due to very good DNA quality. Not too surprising, several of the samples with normal cytology showed Ct values also for the cancer marker regions, but in the range above 37 cycles. Therefore, in contrast to the GynTect analysis of STM samples [13], for using the samples out of LBC medium a threshold for the marker Ct values in relation to the controls was set. Using a delta Ct threshold to the ACHE control region of 9 for the five markers ASTN1, DLX1, ITGA4, RXFP3, and SOX17 and a delta Ct threshold of 10 for ZNF671, we achieved a very good specificity (< 98%) in the NILM group combined with an excellent sensitivity for cancer cases (100%) and a detection rate for the different precancerous stages CIN1, 2 and 3 increasing with grade. This confirmed the results we obtained with samples collected in STM medium [13].

All five cervical cancer samples included in the study were GynTect-positive, each of them for at least four of the six GynTect markers. The high sensitivity for cancer cases was already shown previously analysing 123 (123/123 detected) and 5 (5/5 detected) cases, respectively [12, 13]. A detection rate of > 60% among the CIN3 samples included in the study also confirms results obtained in previous studies [12, 13]. Furthermore, the data show that the GynTect score is related to the histopathologic severity of the lesion. In fact, the higher the CIN grade, the more GynTect markers are detected in the LBC samples. This may reflect the fact that methylation increases with the severity of the lesion.

It is well-known that not all CIN3 lesions proceed to cervical cancer [15, 16]. In several observational studies CIN2/3 short-term regression rates around 30% were reported [17–20]. Very recently, Tainio and colleagues in a meta-analysis summarized CIN2 progression and regression rates of more than 3000 women out of 36 studies [21]. Regression and progression rates ranging from 3 months to up to 60 months were analysed, whereas most data were available for 24 months observation. Overall, a 50% regression rate (11 studies including 1470 women) and an 18% progression rate (9 studies, 1445 women) was observed looking at a 24 months interval. In a subgroup analysis including 1069 women younger than 30 years, regression rates were 60% and only 11% showed progression to higher lesions [21]. One of these studies performed by Loopik et al. demonstrated that among the 211 women < 25 years included in the study, the long-term regression rate of CIN2 lesions was even 71%, whereas the overall progression rate in this study was similar to the other studies (16.6%) [22].

Taking these observations into account with respect to the GynTect-positive results obtained for the three CIN stages, one may hypothesize that only lesions from women tested GynTect-positive may progress to higher grade or cancer while lesions from women tested negative for GynTect are likely to regress to normal. Further studies will show whether regression of CIN lesions correlates with a negative GynTect result.

To compare specificity, sensitivity, PPV and NPV, CINtec Plus was performed for all cytology-abnormal samples plus 59 of the NILM samples. Within this subgroup of randomly chosen NILM cases the CINtec Plus specificity was comparable to GynTect. The cytologically and histopathologically abnormal samples, however, were all positive for the CINtec Plus test. Thus, the test would not allow a differentiation between lesions prone to progression and lesions which may persist or regress. This is well reflected by the PPV (CIN3+) for CINtec Plus with 69.2% compared to GynTect with 74.5%. Other studies using CINtec Plus as triage option showed similar results, even though not all had a 100% sensitivity for CIN1+ samples.

False-positive rates for CINtec Plus reported in the group with normal cytology are in the range of 27–55% [23, 24] and specificity for CIN3+ between 51.3–82.1% [6, 7], being highest in the PALMS trial (94.8%, [7]). CINtec Plus has the ability to specify a triage population out of cytologically abnormal women more precisely than HPV testing [6, 7]. But the GynTect methylation marker panel seems to be more specific with no loss of sensitivity for cancer cases. However, this study only included 5 cancer cases.

HPV genotyping was performed for all samples, and stratification for HPV16/18 positivity leads to a better specificity than HPV in total or CINtec Plus, but still has a lower specificity compared to GynTect for both, CIN2+ and CIN3+. NPV is slightly better for CIN2+ and similar for CIN3+ compared to GynTect. It has to be noted, however, that HPV-positively tested patients who are negative for HPV16/18 – as is the case for one of the five cancer cases included in the study – nonetheless may be detected in the triage following the HPV test, e.g. another cytology.

Cytology results of the 74 CIN lesions from this cohort are difficult to compare to GynTect, HPV or CINtec Plus results since all histologically confirmed CIN lesions have originally been found due to an abnormal cytology result. Therefore, we did only compare GynTect, CINtec Plus and cobas HPV results, because all these were second-line tests after the initial Pap testing in this study.

Methylation markers are discussed as a tool for triage in cervical cancer screening programs, since they have the potential of being more specific than other biomarkers such as p16/Ki-67 (CINtec Plus) or HPV testing, with excellent sensitivity for cervical cancer cases. A recent work from Ochs and colleagues [25] came to the conclusion, that about 50% of all conisations registered at the Hospital in Lucerne, Switzerland, between 2000 and 2014, were performed on women without serious precancerous lesion. This underlines the medical need for more specific diagnostics before referring to conisation.

Meijer et al. established four methylation markers (CADM1, MAL, FAM19A4, mit124–2) with FAM19A4 and mir124–2 being the most promising ones [26–28]. Comparing their clinical performance, sensitivity for cancer and CIN3 seem to be similar to the GynTect markers with 100% cancer detection and around 2/3 CIN3 detection (42.1–100% [27, 28] and 68.8% CIN3 detection [29]). In another study POU4F3 is described as a promising methylation marker, with clinical sensitivity and specificity for CIN3+ of 74 and 89%, respectively [30]. A DNA methylation marker combination PAX1 and ZNF582 is also discussed as a candidate biomarker for triaging suspicious cervical samples [31].

Regarding the specificity, especially for mir124–2 and FAM19A4 a comparison is difficult because no distinction between CIN1 and “no CIN” was made [27, 28]. The GynTect marker panel shows higher specificity for the NILM group with 1.5% detection (GynTect) compared to > 20% for PAX1/ZNF582 or 17.3 and 12.4% for PAX1 and ZNF582 alone [31] or 13% for the panel CADM1/MAL [32]. As well POU4F3 seems to be less specific (specificity CIN3+ 61.4%, with 82.7% sensitivity [33]), even though no data only for the NILM group were reported.

Our results demonstrate that the molecular diagnostic test GynTect based on methylation of the marker regions ASTN1, DLX1, ITGA4, RXFP3, SOX17, and ZNF671 has very good performance using liquid based cytology samples. On one hand, GynTect displayed superior specificity in inconspicuous samples, on the other hand, the test showed excellent sensitivity in detecting the relevant cancer cases. Nevertheless, sample size in this study was small and thus, the power of the study is limited. Further studies have to confirm the results shown in this article.

## Conclusion

In conclusion, in this small sample cohort GynTect shows excellent results if performed on cervical scrape material in liquid-based cytology media, a prerequisite for employing such a test as a triage in screening programs. Compared to CINtec Plus or genotyping for HPV 16 and 18, higher specificity is achieved while still having the option to find all cancer cases and two thirds of high-grade lesions. High-grade lesions tested GynTect-negative may constitute the benign lesions not developing to cancer but instead regressing to normal. The latter has to be shown in prospective trials, of which one is already ongoing, which aims at determining the regression rate of CIN2/3 lesions negative for GynTect in women < 25 years of age.

## Abbreviations

ACHE: Acetylcholinesterase
ASTN1: Astrotactin 1
CADM1: Cell adhesion molecule 1
CIN: Cervical epithelial neoplasia
Ct: Cycle threshold
CxCa: Cervical cancer
DLX1: Distal-less homeobox 1
FAM19A4: Family with sequence similarity 19 member A4
HPV: Human papilloma virus
hrHPV: High-risk HPV
IDS: Iduronate 2-sulfatase
ITGA4: Integrin subunit alpha 4
LBC: Liquid based cytology
MAL: T cell differentiation protein
miR124: MicroRNA 124
NILM: Negative for Intraepithelial Lesion and Malignancy, meaning normal cytlogy
PAX-1: Paired box 1
PCR: Polymerase chain reaction
POU4F3: POU domain, class 4, transcription factor 3
qMSP: real-time methylation specific PCR
RXFP3: Relaxin/insulin like family peptide receptor 3
SOX1: SRY-box 1
SOX17: SRY-box 17
STM: Specimen transport medium, Qiagen
ZNF582: Zinc finger protein 582
ZNF671: Zinc finger protein 671
